# Normative Data for Test of Verbal Fluency and Naming on Ecuadorian Adult Population

**DOI:** 10.3389/fpsyg.2020.00830

**Published:** 2020-05-27

**Authors:** Alberto Rodríguez-Lorenzana, Itziar Benito-Sánchez, Lila Adana-Díaz, Clara Patricia Paz, Tarquino Yacelga Ponce, Diego Rivera, Juan Carlos Arango-Lasprilla

**Affiliations:** ^1^Escuela de Psicología, Universidad de Las Américas, Quito, Ecuador; ^2^Biocruces Bizkaia Health Research Institute, Barakaldo, Spain; ^3^Biomedical Research Doctorate Program, University of the Basque Country (UPV/EHU), Leioa, Spain; ^4^Departamento de Ciencias de la Salud, Universidad Pública de Navarra, Pamplona, Spain; ^5^IKERBASQUE, Basque Foundation for Science, Bilbao, Spain; ^6^Department of Cell Biology and Histology, University of the Basque Country (UPV/EHU), Leioa, Spain

**Keywords:** language, neuropsychological tests, standardization, Ecuador, normative data, verbal fluency, naming

## Abstract

**Objective:** To generate normative data for verbal fluency and naming test in an Ecuadorian adult population.

**Methods:** The sample consisted of 322 healthy adults (18–84 years old) recruited from Quito, Ecuador. The verbal fluency and Boston Naming Test (BNT) were administered as part of a larger comprehensive neuropsychological battery. Multiple linear regression analyses were used to generate the normative data taking into account age, education, and sex.

**Results:** For phonological verbal fluency, results indicated that only education was significantly related to the performance of the letters “A,” “S,” and “M.” However, the performance on the letter “F” was significantly associated with age and education. For semantic fluency, the performance on “animals” was significantly influenced by age, quadratic age, and education, whereas that for “fruits” was explained by quadratic age, education, and sex. The performance on the BNT was significantly influenced by age and education. A Microsoft Excel-based calculator was created to help clinicians to obtain the normative data on this test.

**Conclusion:** This normative data will help neuropsychologist in Ecuador to use these tests both in research and in their clinical practice to improve the diagnosis of cognitive deficits in the population.

## Introduction

Language is involved in most complex activities undertaken by human beings (Roby-Brami et al., 2012). Through language, human beings have been able to preserve cultural meanings over time, thus differentiating human social communication from that of other species (Scott and Schoenberg, 2011; Jodar and Redolar, 2013).

Deficits in one or several of the components and/or functions of language are common in multiple neurological disorders and syndromes (Cuetos, 2012), such as Traumatic Brain Injury (Henry and Crawford, 2004a; Kavé et al., 2011), neurodegenerative diseases (e.g., Alzheimer disease, Parkinson disease, mild cognitive impairment, etc.; Clark et al., 2009; Gardini et al., 2013; Henry and Crawford, 2004b; McDowd et al., 2011), epilepsy (Gleissner and Elger, 2001; Thompson and Duncan, 2005), and stroke (Brady et al., 2001; Kim et al., 2011).

To evaluate language functioning in the presence of neurological alterations, several neuropsychological tests are typically used, for instance, the Boston Aphasia Diagnostic Test (BDAE; Goodglass and Kaplan, 1983), the Western Aphasia Battery (WAB; Kertesz, 1982), Token Test (De Renzi and Vignolo, 1962), the verbal fluency tests, and the Peabody Picture Vocabulary Test (Dunn, 1959). Of these tests, the verbal fluency and the Boston Naming Test (a subtest of the BDAE) are some of the most commonly used by neuropsychologists (Kaplan et al., 1983; Strauss et al., 2006; Lezak, 2012; Fonseca-Aguilar et al., 2015; Olabarrieta-Landa et al., 2016; Arango-Lasprilla et al., 2017).

Verbal fluency is the generation of words based on a specific criterion (letters, categories, actions, etc.). Within research and clinical practice, the most commonly used verbal fluency tests are phonological and semantic (Strauss et al., 2006; Marino and Alderete, 2010; Lezak, 2012). Phonological fluency requires executive functioning and the activation of areas within the frontal lobe, whereas semantic fluency taps into lexical access and vocabulary and requires participation of the temporal lobe (Olabarrieta-Landa et al., 2017). The assessment of this test must comply with certain rules including time limits for word generation, instructions about the types of words that must be evoked and/or omitted, and specific scoring guidelines (Olabarrieta-Landa et al., 2017).

In Spanish-speaking countries, numerous studies have been conducted to obtain normative data of verbal fluency tests, including Spain (Peña-Casanova et al., 2009b; Casals-Coll et al., 2013), Bolivia, Chile, Cuba, El Salvador, Guatemala, Honduras, México, Paraguay, Perú, Puerto (Olabarrieta-Landa et al., 2015a), Argentina (Marino and Alderete, 2010; Olabarrieta-Landa et al., 2015a), and Colombia (Olabarrieta-Landa et al., 2015c). The results of these studies with Spanish speakers indicate that performance on most verbal fluency tests is influenced by education, with higher schooling associated with better performance (Marino and Alderete, 2010; Casals-Coll et al., 2013; Olabarrieta-Landa et al., 2015a, b). Regarding gender, no significant differences were found in a study conducted in Spain (Casals-Coll et al., 2013) or in the study by Olabarrieta-Landa et al. (2015a) involving various Latin American (LA) countries. Gender differences were found only by Marino and Alderete (2010) in an Argentine population, with better performance by men on certain tests of semantic fluency (number of animals, number of tools) and phonological fluency (letter P, total score).

Research exploring age effects on verbal fluency in Spanish speakers has yielded mixed results. Regarding phonological fluency, in a study conducted by Olabarrieta-Landa et al. (2015a), performance worsened with increasing age in the majority of countries in Latin America; however, this effect was not evident in countries such as Argentina, Paraguay, and Guatemala for the letters “F,” “A,” and “S” and in Honduras only for the letter “F.” Regarding sematic fluency, Olabarrieta-Landa et al. (2015a) found that performance worsened with increasing age in all the LA countries studied. Nevertheless, Marino and Alderete (2010) found older age associated with better performance on the semantic fluency category tools in Argentina. These mixed results have been also found in two studies in Spain. For instance, Peña-Casanova et al. (2009a) in a sample of 346 healthy participants between 50 and 94 years old found a significant relationship between age and verbal fluency, but Casals-Coll et al. (2013) in a sample of 179 healthy participants 18 to 49 years old did not find this relationship.

The competence of naming linguistic concepts from visual stimuli involves the ability to integrate different cognitive components, such as perceptual recognition, semantic memory, and the lexical phonological output store (Fernández-Blázquez et al., 2012). The neuropsychological evaluation of naming is usually characterized by using tasks in which the evaluated person is asked to indicate the name of the object that is presented by means of a drawing (confrontation naming test; Harry and Crowe, 2014). The Boston Naming Test (BNT; Kaplan et al., 1983) is one of the most commonly utilized tests. There are different versions of this test, with an original version that consists of 60 items and reduced versions varying between 11 and 30 items. Normative studies in Spanish-speaking countries for the BNT show significant positive relationships between test performance and education (Aranciva et al., 2012; Fernández-Blázquez et al., 2012; Olabarrieta-Landa et al., 2015b). The association of test performance with age has been mixed. Olabarrieta-Landa et al. (2015b) found a negative correlation in certain Spanish-speaking countries, while Fernández-Blázquez et al. (2012) and Peña-Casanova et al. (2009a) found that this correlation exists only after 50 years of age. Regarding gender, Aranciva et al. (2012) found no significant differences, whereas Olabarrieta-Landa et al. (2015b) found better performance on the BNT by men in Mexico, Argentina, Chile, Cuba, Guatemala, and Bolivia.

In recent years, country-specific normative data have become available for neuropsychological tests commonly used in the majority of Spanish-speaking countries. However, country-specific data for Ecuador are lacking in the literature. The result is that professionals must interpret raw score or use norms from populations from other Spanish-speaking countries (Arango-Lasprilla and Rivera, 2015; Olabarrieta-Landa et al., 2015a, c). The aim of this study is to provide normative data for two of the most commonly used tests to measure verbal fluency and naming in the Ecuadorian adult population.

## Materials and Methods

### Participants

The sample consisted of 358 healthy individuals who were recruited from Quito, Ecuador. Participants’ ages ranged from 18 to 84 years (mean = 41.3, SD = 18.2). Education ranged from 2 to 25 years (mean = 13.2, SD = 4.6). The majority was women (54.04%), and the sample was primarily urban (82.8%). The sampling strategy was determined by taking into consideration factors such as literacy level; percentage of people with primary, secondary, and post–secondary education; and age distribution. It is important to note that generating normative data with a population sample from Quito could have some problems, such as overestimation/underestimation of normative data from other population areas of Ecuador. However, Quito is one of the most representative cities of Ecuador, with more than 2.8 million residents and included people from all the cities of the country. On the other hand, the sampling error in this study is ≈0.055 (accuracy level ≈94.5%), which allows us to make adequate inferences. The maximum error was established using classical estimation assuming infinite (very large) population sizes, where the case of maximum uncertainty was assumed (π = 1–π = 0.5) and a confidence interval of 95%. The demographic characteristics (age, education, and sex) can be found in Table 1.

**TABLE 1:** Demographic characteristics of the sample.

		Age		Education		Sex	
		
						Woman	Man
Age group	*n*_*i*_	Mean	SD	Mean	SD	*n*	*n*
20 ± 2 year	41 (12.7%)	20.1	1.4	12.7	3.1	22	19
25 ± 2 year	62 (19.3%)	24.9	1.4	13.6	3.7	26	36
30 ± 2 year	30 (9.3%)	29.8	1.5	15.0	4.4	19	11
35 ± 2 year	30 (9.3%)	35.0	1.4	14.1	5.2	17	13
40 ± 2 year	26 (8.1%)	39.9	1.4	12.2	4.1	14	12
45 ± 2 year	26 (8.1%)	44.6	1.1	13.3	4.2	14	12
50 ± 2 year	20 (6.2%)	50.1	1.6	13.2	4.7	14	6
55 ± 2 year	20 (6.2%)	55.7	1.4	14.1	6.2	13	7
60 ± 2 year	16 (5.0%)	60.3	1.2	14.5	4.6	9	7
65 ± 2 year	13 (4.0%)	64.5	1.5	13.4	6.0	7	6
70 ± 2 year	11 (3.4%)	68.5	0.7	11.8	5.0	6	5
75 ± 2 year	11 (3.4%)	75.2	1.4	11.5	6.8	5	6
>78 year	16 (5.0%)	81.2	1.9	9.6	3.7	8	8
Total	322	41.3	18.2	13.2	4.6	174	148

*SD = standard deviation.*

To be eligible to participate, individuals had to meet the following inclusion criteria: (a) were between 18 and 84 years of age, (b) were born and currently live in Ecuador, (c) spoke Spanish as their native language, (d) had completed at least 1 year of formal education, (e) were able to read and write at the time of evaluation, (f) scored ≥23 on the Mini-Mental State Examination (MMSE; Villaseñor-Cabrera et al., 2010), (g) scored ≤4 on the Patient Health Questionnaire 9 (PHQ-9, Kroenke et al., 2001), (h) scored ≥90 on the Barthel Index (Mahoney and Barthel, 1965), (i) have no history of diagnosed neurological or psychiatric conditions, (j) have no history of alcohol abuse or other psychotropic substances, (k) no history of systemic diseases that affect cognition (e.g., diabetes mellitus), (l) not regularly using pain medications or others that may affect cognitive functioning, (m) not having severe vision and/or hearing deficits, and (n) not having a history of learning or neurodevelopmental problems. All test participants were volunteers who did not receive financial compensation for participation.

The final sample was 322 because 36 participants were excluded (18 score < 23 in MMSE; 9 score > 4 in the PHQ-9; and 9 score < 90 in the Barthel Index). Participants’ mean MMSE score was 28.65 (range = 23–30; SD = 1.457). For the PHQ-9, the mean was 1.74 (range = 0–4; SD = 1.215). Finally, for the Barthel Index, the mean was 100, because all participants score the highest.

### Procedure

The present study was conducted as part of a larger study to generate normative data for a series of neuropsychological measures in Spanish-speaking populations (Guàrdia-Olmo et al., 2015; Rivera and Arango-Lasprilla, 2017). The ethics committee of the Universidad San Francisco de Quito approved the study.

Participants were volunteers from the community recruited through announcements distributed in local business, community centers, and the university. All the persons who showed interest in participating were contacted by one member of the research team, who explained the characteristics of the study and answered any questions that the person might have. People who agreed to participate signed the informed consent, and then their sociodemographic data were collected; it was followed by the application of the PHQ-9, the Barthel Index, and the MMSE to verify inclusion and exclusion criteria. A comprehensive battery of neuropsychological tests including those who measured verbal fluency and naming was administered to those participants who met the inclusion criteria. The administration of the tests was carried out by undergraduate psychology students under the supervision of a neuropsychologist in the facilities of the Universidad Las Américas. The assessment lasted between 80 and 120 min. Data collection started in May 2017 and ended in March 2019.

### Measures

#### Patient Health Questionnaire 9 (PHQ-9)

It is a nine-item scale that assesses the presence of depressive symptoms based on the criteria of the *Diagnostic and Statistical Manual of Mental Disorders* (Baader et al., 2012).

#### Mini-Mental State Examination (MMSE)

It is a screening test that assesses cognitive function and is sensitive to its deterioration. The test examines five major areas of mental functioning: orientation, retention, attention and calculation, memory, and language. A score of 23 or less indicates cognitive decline (Tombaugh et al., 1996).

#### Barthel Index for Activities of Daily Living

It provides measures of the individual’s performance, on 10 activities of daily life (feeding, bathing, grooming, dressing, bowels, bladder, toilet use, transfers, mobility, and stairs; Shah et al., 1989). A total score between 0 and 20 suggests total dependence; 21–60, severe dependence; 61–90, moderate dependence; 91–99, mild dependence; and 100, independence.

#### Verbal Fluency Tests (VFT)

The aim of the phonological VFT (P-VFT) is to produce as many words as possible that begin with a specified letter within 60 s. For this study, the following letters were selected: F, A, S, and M. The letters F, A, and S correspond to the original stimuli of the test and are the most frequently used letters in the literature (Olabarrieta-Landa et al., 2017). The letter M was included because it is one of the letters with higher frequency in Spanish language (Artiola et al., 1999; Peña-Casanova et al., 2009b). In Semantic Verbal Fluency Test (S-VFT), the participant is required to produce as many words as possible belonging to a certain category in 60 s (in this study, animals and fruits). The total score consisted of the total correct answers for each letter or category. Proper names, intrusions, and perseverations were not allowed. In case of supracategory, both supracategory (e.g., bird) and examples of it (e.g., crow, sparrow) were allowed. For the exact instructions and scoring rules, refer to Strauss et al., 2006 (p. 499).

#### Boston Naming Test (BNT)

The BNT present 60 pictures (Standard version) or 15 pictures (Short version) in order of increasing difficulty. The aim of the task is to denominate each picture. If the correct answer is not given spontaneously, the examiner provides a semantic clue (in case of misrecognition error) or phonological clue (when the semantic clue is still not enough, or during the spontaneous response there has been an error that is not a misrecognition error). For this study, the Spanish Standard and Short versions of the BNT (Kaplan et al., 2005) were used, and the total score was considered as the sum of correct spontaneous answers plus correct answers followed by a semantic clue.

### Statistical Analyses

#### Exploratory Data Analysis

Pearson correlations between the VFT scores (including letters F, A, S, M; categories animals, fruits), BNT scores (Standard and Short), and the sociodemographic (age, education and sex) variables were computed. Mean total P-VFT score was calculated summing up F, A, S, and M total scores and dividing it by 4: [(F + A + S + M)/4]. Mean total S-VFT score was calculated summing up animals and fruits total scores and dividing it by 2: [(animals + fruits)/2].

#### The Effects of Demographic Variables and the Derivation of Normative Data

Verbal fluency (F, A, S, M, and mean P-VFT scores), semantic fluency (animals, fruits, and mean S-VFT scores), and BNT (Standard and Short scores) scores were computed separately evaluating the effects of demographic variables on each score by means of multiple linear regression analyses. The full regression models included as predictors the following: age, age^2^, education, education^2^, sex, and all two-way interactions between these variables. Age and education were centered (= calendar age in years – mean age in the sample; education in years – mean education in the sample) before computing the quadratic age and education to avoid multicollinearity (Kutner et al., 2005). Quadratic age and years of education were added into the full model to allow for quadratic effects between these independent variables and the scores of each test. Sex was dummy coded as man = 1 and woman = 0. The full regression model can be formally described as follows:

$$Y_{i}=B_{0}+B_{1}\cdot {\left(\text{Age}-41.3\right)}_{i}+B_{2}\cdot {\left(\text{Age}-41.3\right)}_{i}^{2}\,\;+B_{3}\cdot {\left(\text{Education}-13.2\right)}_{i}+B_{4}\cdot {\left(\text{Education}-13.2\right)}_{i}^{2}\,\;+B_{5}\cdot {\text{Sex}}_{i}+B_{k}\cdot {\text{Interactions}}_{i}+\varepsilon_{i},$$

with the term Interactions_*i*_ referring to all two-way interactions between the fixed effects. The predictors that were not statistically significant in the multiple regression model were removed, and the reduced model was readjusted. A Bonferroni-corrected α level of 0.005 (= 0.05/10) was used. No predictor was removed if it was also included in a higher-order term in the model (Aiken et al., 1991). For all multiple linear regression models, the following assumptions were evaluated: multicollinearity (variance inflation factor [VIF] ≤ 10), homoscedasticity (participants were grouped into quartiles of the predicted scores, and the Levene test was applied on the residuals), normality of the standardized residuals (Kolmogorov–Smirnov test), and the existence of influential values assess (calculation of the maximum Cook’s distance, and subsequently related to an *F*(*p*,*n*−*p*) distribution; Kutner et al., 2005). An α level of 0.005 was used in all analyses.

The normative data that fit for the demographic variables were established through a four-step procedure, using the final regression model obtained at the end of the procedure (Rivera et al., 2019, 2020): (a) The expected test score (Ŷ_*i*_) is computed based on the fixed effect parameter estimates of the established final regression model: Ŷ_*i*_ = B_0_ + B_1_X_1*i*_ + B_2_X_2*i*_ + … +B_k_X_k*i*_. (b) To obtain the residual value (*e*_*i*_), a subtraction between the raw score of the neuropsychological test (*Y*_*i*_) and the predicted value previously calculated was performed (Ŷ_*i*_), as shown in the following formula: *e*_*i*_=*Y*_i_ – Ŷ_*i*_. (c) Using the residual standard deviation (*SD*_*e*_) value provided by the regression model (Table 2), residuals were standardized: *z*_*i*_ = *e*_*i*_/*SD*_*e*_. (d) Finally, using the standard normal cumulative distribution function, the exact percentile corresponding to the *z* score previously calculated was obtained (if the model assumption of normality of the residuals was met in the normative sample), or via the empirical cumulative distribution function of the standardized residuals (if the standardized residuals were not normally distributed in the normative sample; for further information about BNT Standard score, see Supplementary Appendix 1). This four-step process was applied to the VFT (F, A, S, M, and mean P-VFT, animals, fruits, and mean S-VFT) scores and BNT (Standard and Short total score) scores separately.

**TABLE 2:** Standard deviation (residual) for final multiple linear regression models.

Test	Predicted value	Std. deviation
F	All values	3.749
A	All values	3.848
S	All values	4.001
M	All values	4.089
Mean P-VFT	All values	3.244
Animals	All values	4.308
Fruits	All values	3.230
Mean S-VFT	All values	3.253
BNT standard	<47.414	7.728
	47.415–50.740	7.197
	50.741–53.124	5.426
	>53.124	4.601
BNT short	All values	1.994

Adjusted *R*^2^ values are provided for all final models. All analyses were performed using SPSS version 23 (IBM Corp., 2015).

## Results

### Exploratory Data Analysis

Table 3 shows the intercorrelation between all the scores (letters F, A, S, M, animals and fruits categories, Standard and Short BNT) and the sociodemographic variables (age, education and sex).

**TABLE 3:** Correlations between all VFT scores and demographic variables.

	*F*	*A*	*S*	*M*	Animals	Fruits	BNT standard	Age	Education	Sex
*F*	−	−	−	−	−	−	−	−0.216**	0.326**	0.003
*A*	0.534**	−	−	−	−	−	−	−0.130*	0.361**	0.014
*S*	0.634**	0.540**	−	−	−	−	−	−0.139*	0.358**	–0.054
*M*	0.667**	0.644**	0.713**	−	−	−	−	–0.106	0.362**	–0.019
Animals	0.446**	0.487**	0.492**	0.519**	−	−	−	−0.382**	0.440**	0.074
Fruits	0.350**	0.378**	0.405**	0.446**	0.564**	−	−	−0.287**	0.252**	−0.203**
BNT standard	0.348**	0.348**	0.354**	0.407**	0.437**	0.291**	−	−0.267**	0.448**	0.138*
BNT short	0.348**	0.343**	0.319**	0.383**	0.445**	0.303**	0.763**	−0.274**	0.467**	0.189**

**p < 0.001; *p < 0.05.

The intercorrelation between letters F, A, S, and M scores can be seen with age (all *r* ≥ —0.130—; *p* < 0.001) and education (all *r* ≥ 0.326; *p* < 0.001). Sex was no significant with any score. In view of the high correlations between all phonological VFT scores (*r* ≥ 0.534; *p* < 0.001), a mean P-VFT total score [(F + A + S + M)/4] was calculated, and normative data were provided for this overall test score summary metric.

For animals and fruits categories, the intercorrelation was with age (*r* ≥ —0.287—; *p* < 0.001), education (*r* ≥ 0.252; *p* < 0.001), and sex (*r* = −0.203; *p* < 0.001; in fruits). In view of the high correlations between all semantic VFT scores (*r* ≥ 0.564; *p* < 0.001), a mean S-VFT total score [(animals + fruits)/2] was calculated, and normative data were provided for this overall test score summary metric.

Finally, the intercorrelation between BNT Standard and Short scores was with age (all *r* ≥ —0.267—; *p* < 0.001), education (all *r* ≥ 0.448; *p* < 0.001), and sex (all *r* ≥ 0.138; *p* < 0.05).

### Model Assumptions

The assumptions of multiple linear regression analysis were fulfilled for all final models. There was no multicollinearity (VIF values in all models were at most 1.997, and therefore well below the threshold value = 10; collinearity tolerance values did not exceed the value of 1) or influencing cases (the maximum distance Cook’s value was 0.182; relating this value to an *F*_(__3_,_319__)_ distribution produces the 9^th^ percentile value, which is well below the threshold percentile value = 50). Levene test suggested that there was homoscedasticity in all models except for the standard BNT score. The standardized residuals of all models were normally distributed (as evaluated with the Kolmogorov–Smirnov test).

### The Effects of Demographic Variables

#### Phonological Verbal Fluency

The final multiple linear regression models for letters F, A, S, M, and mean P-VFT scores were significant (Table 4). Letters F, A, S, M, and the mean P-VFT total score were positively influenced by education, so those with higher education generated more words in each letter. Letter F score was also negatively influenced by age, showing that young people have better performance. The amount of variance (adjusted for the number of predictors in the final model; adjusted *R*^2^) explained by these predictors was 13% for letters F, A, and M; 12% for letter S; and 17% for the mean P-VFT total score. A, S, M, and P-VFT scores were not affected by sex and all two-level interactions.

**TABLE 4:** Final multiple linear regression models for Phonological VFT.

Letter		*B*	Std. error	β	*t*	Sig.	Adjusted *R*^2^
*F*	(Intercept)	11.208	0.210		53.466	<0.001	0.132
	Age	–0.040	0.012	–0.178	–3.390	0.001	
	Education	0.267	0.046	0.303	5.784	<0.001	
*A*	(Intercept)	12.072	0.215		56.193	<0.001	0.127
	Education	0.325	0.047	0.361	6.920	<0.001	
*S*	(Intercept)	11.429	0.223		51.178	<0.001	0.125
	Education	0.335	0.049	0.358	6.855	<0.001	
M	(Intercept)	12.360	0.228		54.158	<0.001	0.128
	Education	0.347	0.050	0.362	6.948	<0.001	
Mean P-VFT	(Intercept)	11.767	0.181		64.978	<0.001	0.170
	Education	0.324	0.040	0.415	8.168	<0.001	

*VFT = Verbal Fluency Test.*

#### Semantic Verbal Fluency

**FIGURE 1:**
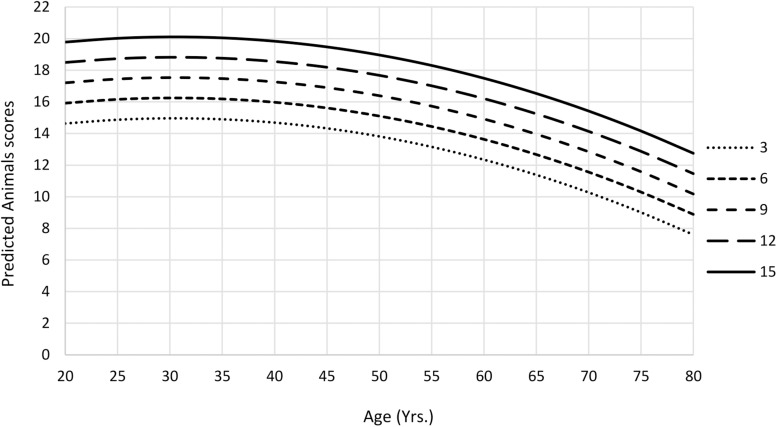
Predicted mean Animals scores as a function of age and education from Ecuadorean sample.

**FIGURE 2:**
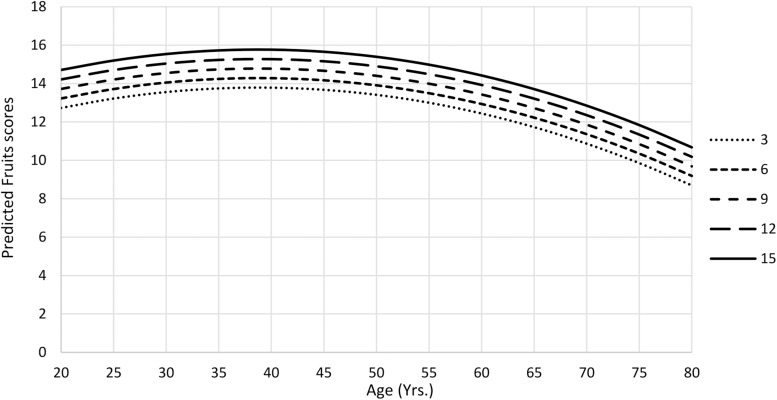
Predicted mean Fruits scores as a function of age and education from Ecuadorean sample.

The amount of variance explained after adjusted for the number of predictors in animals final model was 32%; for fruits model, 25%; and for mean S-VFT total scores model, 33% (Table 5). The animals and fruits categories and the mean S-VFT total scores were negatively affected by quadratic age effect, showing a curvilinear pattern of the scores according to age, with a fall after the age of 40 years (Figures 1, 2). All scores were affected by education, so that those with higher education generated more words in each category. Fruits score was also negatively affected by sex, with women obtaining better scores than men.

**TABLE 5:** Final multiple linear regression models for Semantic VFT.

Categories		*B*	Std. error	β	*t*	Sig.	Adjusted *R*^2^
Animals	(Intercept)	18.984	0.350		54.171	<0.001	0.319
	Age	–0.065	0.016	–0.225	–3.990	<0.001	
	Age^2^	–0.003	0.001	–0.191	–3.363	0.001	
	Education	0.429	0.054	0.374	7.976	<0.001	
Fruits	(Intercept)	17.040	0.302		56.485	<0.001	0.254
	Age	–0.015	0.012	–0.072	–1.220	0.223	
	Age^2^	–0.003	0.001	–0.343	–5.716	<0.001	
	Education	0.165	0.041	0.201	4.058	<0.001	
	Sex	–1.585	0.369	–0.210	–4.302	<0.001	
Mean S-VFT	(Intercept)	17.695	0.265		66.860	<0.001	0.332
	Age	–0.038	0.012	–0.171	–3.072	0.002	
	Age^2^	–0.003	0.001	–0.301	–5.333	<0.001	
	Education	0.286	0.041	0.327	7.036	<0.001	

*VFT = Verbal Fluency Test.*

#### Boston Naming Test

**FIGURE 3:**
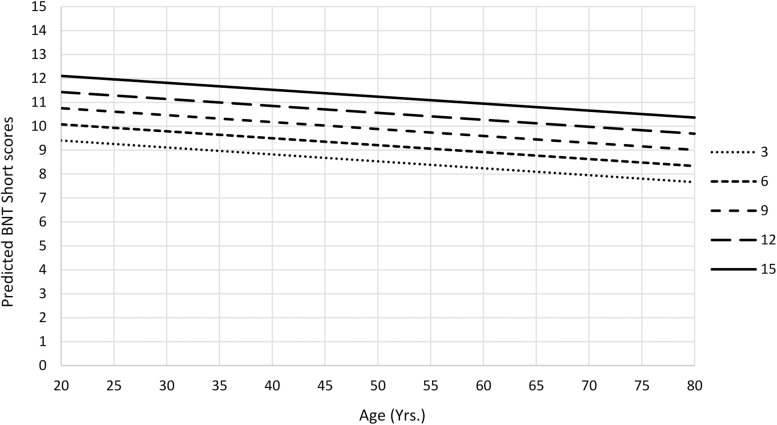
Predicted mean BNT Short scores as a function of age and education from Ecuadorean sample.

The final multiple linear regression models for BNT Standard and BNT Short scores were significant (Table 6). The amount of variance explained after adjusting for the number of predictors in BNT Short final model was 24%, and for BNT Standard, final model was 26%. Both BNT Short and Standard total scores were negatively influenced by age and increased linearly as a function of education, showing young and more educated people have better performance (Figure 3).

**TABLE 6:** Final multiple linear regression models for BNT.

BNT		*B*	Std. error	β	*t*	Sig.	Adjusted *R*^2^
Standard	(Intercept)	50.117	0.356		140.860	<0.001	0.243
	Age	–0.088	0.020	–0.218	–4.435	<0.001	
	Education	0.677	0.079	0.423	8.618	<0.001	
Short	(Intercept)	11.082	0.112		99.228	<0.001	0.263
	Age	–0.029	0.006	–0.222	–4.601	<0.001	
	Education	0.225	0.025	0.441	9.130	<0.001	

*BNT = Boston Naming Test.*

### Normative Data Calculator

The four-step normative procedure explained above allows determining an exact percentile value for VFT and BNT test scores. However, this method can be prone to human error due to the number of required computations by hand. To enhance user-friendliness, the authors created a calculator in Microsoft Excel that conducts all required computations. The clinician simply fills in raw VFT and BNT scores and demographic characteristics (i.e., age, education, and sex), and the software automatically computes the standardized residuals and their corresponding exact percentile values. This tool is freely available for all users and may be downloaded at https://neuropsychologylearning.com/datos-normativos-archivos-descargables/.

## Discussion

The aim of the current study was to generate normative data for the Verbal Fluency Test and for the Boston Naming Test for an Ecuadorian adult population, while controlling the effect of sociodemographic variables, such as age, sex, and level of education, on performance. In addition, this study presents a calculator of normative data, which simplifies the procedure to obtain the norms. This calculator allows reducing time and possible human errors when data are visualized in tables (Ramírez et al., 2012).

Linear regression models for phonological fluency explained approximately 13% of the variance for all letters considered. For letters “A,” “S,” and “M,” the results were similar to those found by Casals-Coll et al. (2013) in Spain with young adults (18–49 years old) and Olabarrieta-Landa et al. (2015a) in Argentina, Guatemala, and Paraguay with adults in a wider age range from 18 to 95 years, in which performance was only associated with education; scores improved as level of education increased. Age was not a significant predictor of performance. However, it differs from the study by Peña-Casanova et al. (2009a) carried out in Spain with older adults (50–94 years) and from the study by Olabarrieta-Landa et al. (2015a) in other LA countries such as Puerto Rico, Peru, Mexico, Honduras, El Salvador, Cuba, Chile, and Bolivia. The differences with Peña-Casanova et al. (2009a) study may be explained because of the different letters used in the study. While Peña-Casanova and colleagues used the letters “M,” “P,” and “R,” the most appropriate for Spanish vocabulary, in the present study the letters “F,” “A,” “S,” and “M” (the most frequently used letters in the literature) were used. Furthermore, the α level to avoid type I error differs among studies. In this study, the threshold was adjusted to 0.005 (Van Der Elst et al., 2006; Rivera et al., 2019), whereas in Peña-Casanova et al. (2009a), it was at 0.05.

Finally, for letter “F,” the results are consistent with those found by Olabarrieta-Landa et al. (2015a) in Bolivia, Chile, Cuba, El Salvador, Mexico, Peru, and Puerto Rico, and by Peña-Casanova et al. (2009a) in which both age and education were positively related to performance on this letter. Nevertheless, in other LA countries such as Argentina, Guatemala, Honduras, and Paraguay, the effect was only explained by education but not by age. No gender effect was noted in the models, which was generally consistent with prior research with the exception of Marino and Alderete (2010) conducted in Argentina, in which sex and education significantly affected performance.

Models for semantic fluency explained 25% to 32% of the variance. For “animals,” the variables age, quadratic age, and education significantly explained the performance (32% of the variance). The results are consistent with the study of Olabarrieta-Landa et al. (2015a), for several LA countries, Argentina, Bolivia, Chile, Cuba, El Salvador, Guatemala, Honduras, Mexico, Paraguay, Peru, and Puerto Rico, in which scores worsen with increasing age, whereas scores improved when the number of years of education increased; in that study, quadratic age was not included in the model. Gender did not significantly explain performance; however, Marino and Alderete (2010) found that in Argentina this variable jointly with education was associated with performance; men with more years of education had better performance scores than women. Scores for “fruits” were significantly associated by quadratic age, education, and sex. People with greater levels of education and younger named a greater number of fruits. However, after age 40 years, the number of named fruits decreased, which is explained by the quadratic distribution of the scores. Women presented better performance in this category than did men. The influence of education is consistent with previous studies (Marino and Alderete, 2010; Casals-Coll et al., 2013; Olabarrieta-Landa et al., 2015a). However, the present study is one of the first studies in Spanish-speaking countries that included in the model the quadratic age, which enhanced the interpretability of the results.

The BNT was used for the assessment of naming. The results obtained in the present study were similar to those found in a previous study conducted in Latin America in which BNT scores decreased linearly as a function of age and improved as number of years of education increased in several countries, such as Chile, Cuba, El Salvador, Guatemala, Mexico, and Puerto Rico (Olabarrieta-Landa et al., 2015b). These results are also consistent with those of the study conducted by Fernández-Blázquez et al. (2012), although with older adults in Spain. Only the study of Aranciva et al. (2012) found no significant relationship between age and performance in this test; nevertheless, it can be an effect of the limited range of age (18–49 years) of the population in that study. In the present study, gender was not a significant predictor of the performance on BNT scores, similarly to previous studies (Aranciva et al., 2012; Fernández-Blázquez et al., 2012).

The results of the present study have important implications. To our knowledge, this is the first study in Ecuador that presents normative data for two of the most common neuropsychological tests that are used to assess verbal fluency and naming functions. This referential data will allow clinicians to have more precise interpretation of scores and the variables to consider when these tests are used in this population. In the past, the interpretation of scores for these tests in this country was done using raw data or normative data from other countries such as United States, United Kingdom, Canada, and Spain, which possibly resulted in biases and identification of false-positive cases. Suppose we need to find the percentile score for an Ecuadorian man, who is 60 years old, has 5 years of education, and scored 12 on animals. Based on the normative data done by Tombaugh et al. (1999) for Canada, this person would have obtained an adjusted *z* score of −0.705 (percentile 24). With the normative data obtained by Peña-Casanova et al. (2009a) for Spain, the Neuronorma Scaled Score would have been of 7 (percentile 11–18). If we compared with other LA Spanish-speaking countries, such as Mexico or Paraguay (Olabarrieta-Landa et al., 2015a), the adjusted *z* scores would have been of −0.927 (percentile 18) for Mexico. With the norms generated in this study, this person would have obtained an adjusted *z* score of −0.279 (percentile 39). The previous norms from other countries placed the individual in percentiles much lower than the one obtain using the country-specific norms presented here. This is only one example to illustrate the potential biases of using other norms to interpret raw scores.

Moreover, compared with other studies that have been done in Lain America, this study presents a novel methodology for the calculation of the normative data, which lead to more accurate data than in previous studies. First, to avoid multicollinearity within the multiple regression model, age and education were centered. Second, quadratic age and education were included in the model, which allowed to test whether there is an age or education range in which the performance starts to decrease. Third, all the possible interactions between the variables were tested to determine which variables significantly explain the performance in each test.

### Limitations

The results of this study should be interpreted in light of the following limitations. First, the data were collected in the Metropolitan District of Quito, which is one of the most representative cities of Ecuador, which have residents from all the cities of the country. Generating normative data with a population sample from Quito can overestimate/underestimate the normative data in other population areas of Ecuador. Future studies might include participants from rural areas and other different regions of the country in order to have a more representative sample of the Ecuadorian population. Second, another variable that was not considered in the study was bilingualism, which previous studies have shown to influence cognitive performance. Although in Ecuador, Spanish is the official language, approximately 34% of the population is indigenous, and there are 18 indigenous native languages (Haboud et al., 2016). Then, it is relevant for future studies to assess the level of bilingualism on these populations and to determine its influence on the results. Third, in this study, the number of years of education was considered, and other variables related to the quality of education were not included on the data collection variables. Future studies might include this variable and to determine its influence on the test performance. Finally, this study only presented normative data in healthy Ecuadorian individuals. Future studies should be done with clinical populations (e.g., Alzheimer, traumatic brain injury, stroke, etc.) in order to determine the specific cutoff scores on these tests by group.

### Conclusion

The purpose of this study was to develop normative data for two of the most common neuropsychological tests used by neuropsychologist in Ecuador to measure verbal fluency and naming in adult population. Results show that education had a significant positive effect on the performance of letters “A,” “S,” and “M”; the performance improved when the number of years of education increased. For letter “F,” in addition to education, the age was also significantly associated with the performance, such as the older that person is, the worse the performance. For semantic fluency, the performance on “animals” improved when the person has a greater number of years of education and when the person is younger. However, when including age^2^, it is evident that the number of fruits named increased until the age 40 years, and then it starts to decrease. Education and age had similar association in regard to the performance on “fruits”; however, women named more fruits than did men. The BNT scores were significantly explained by age and education.

Verbal fluency and naming problems are very common symptoms reported in a variety of patients with neurological disorders. Therefore, it is expected that this normative data will be widely used and contributed to improve the clinical practice of neuropsychologists in Ecuador, helping clinicians to avoid the malpractice of using raw scores or normative data from other countries when conducting neuropsychological evaluations.

## Data Availability Statement

Research data are not shared publicly due to stipulations made by the research ethics committee at the time of approval regarding the storage and confidentiality of patient data. For requests please contact the corresponding author.

## Ethics Statement

The studies involving human participants were reviewed and approved by the Ethics Committee of the Universidad San Francisco de Quito. The patients/participants provided their written informed consent to participate in this study.

## Author Contributions

AR-L: conceptualization, investigation, resources, writing – original draft, project administration, and funding acquisition. IB-S: formal analysis, writing – review and editing. LA-D: conceptualization, investigation, resources, writing – original draft, and funding acquisition. CP: conceptualization, investigation, resources, writing – original draft, and funding acquisition. TY: conceptualization, investigation, resources, writing – original draft, project administration, and funding acquisition. DR: methodology, formal analysis, writing – review and editing. JA-L: conceptualization, methodology, writing – review and editing, and supervision.

## Conflict of Interest

The authors declare that the research was conducted in the absence of any commercial or financial relationships that could be construed as a potential conflict of interest.
