# Supplementary material for: Normative Data for Test of Verbal Fluency and Naming on Ecuadorian Adult Population
Source: Front Psychol. 2020 May 27;11:830. doi: 10.3389/fpsyg.2020.00830 (PMC7267034; doi:10.3389/fpsyg.2020.00830)
Supplement: Supplementary file 1 [file Data_Sheet_1.docx]

Appendix 1. Adjusted Z score for BNT Standard score.

| **Percentile** | **Z score** | **Percentile** | **Z score** | **Percentile** | **Z score** | **Percentile** | **Z score** |
| --- | --- | --- | --- | --- | --- | --- | --- |
| ≤1 | -3.1045 | 26 | -0.5197 | 51 | 0.1998 | 76 | 0.7837 |
| 2 | -2.6977 | 27 | -0.5038 | 52 | 0.2089 | 77 | 0.7904 |
| 3 | -2.3635 | 28 | -0.4361 | 53 | 0.2555 | 78 | 0.8014 |
| 4 | -2.2416 | 29 | -0.4272 | 54 | 0.2805 | 79 | 0.8316 |
| 5 | -2.0432 | 30 | -0.3930 | 55 | 0.3103 | 80 | 0.8464 |
| 6 | -1.7035 | 31 | -0.3485 | 56 | 0.3315 | 81 | 0.8655 |
| 7 | -1.4911 | 32 | -0.3412 | 57 | 0.3556 | 82 | 0.8975 |
| 8 | -1.4429 | 33 | -0.3320 | 58 | 0.3689 | 83 | 0.9620 |
| 9 | -1.3880 | 34 | -0.2978 | 59 | 0.3969 | 84 | 0.9989 |
| 10 | -1.3315 | 35 | -0.2485 | 60 | 0.4172 | 85 | 1.0451 |
| 11 | -1.1931 | 36 | -0.2326 | 61 | 0.4311 | 86 | 1.0638 |
| 12 | -1.1282 | 37 | -0.2262 | 62 | 0.4647 | 87 | 1.1048 |
| 13 | -1.1070 | 38 | -0.2131 | 63 | 0.4715 | 88 | 1.1161 |
| 14 | -1.0718 | 39 | -0.1811 | 64 | 0.4829 | 89 | 1.1356 |
| 15 | -1.0280 | 40 | -0.1465 | 65 | 0.5213 | 90 | 1.1475 |
| 16 | -0.9845 | 41 | -0.1080 | 66 | 0.5470 | 91 | 1.1692 |
| 17 | -0.9408 | 42 | -0.0574 | 67 | 0.5695 | 92 | 1.2108 |
| 18 | -0.8937 | 43 | -0.0417 | 68 | 0.6218 | 93 | 1.2458 |
| 19 | -0.8341 | 44 | -0.0171 | 69 | 0.6281 | 94 | 1.2563 |
| 20 | -0.8093 | 45 | 0.0035 | 70 | 0.6389 | 95 | 1.2831 |
| 21 | -0.7939 | 46 | 0.0413 | 71 | 0.6563 | 96 | 1.3166 |
| 22 | -0.6962 | 47 | 0.0674 | 72 | 0.6718 | 97 | 1.3457 |
| 23 | -0.6563 | 48 | 0.1506 | 73 | 0.7107 | 98 | 1.4537 |
| 24 | -0.5808 | 49 | 0.1711 | 74 | 0.7424 | 99 | 1.4929 |
| 25 | -0.5661 | 50 | 0.1873 | 75 | 0.7673 | >99 | 2.0165 |
